# Inferring position of motor units from high-density surface EMG

**DOI:** 10.1038/s41598-024-54405-1

**Published:** 2024-02-15

**Authors:** Jonathan Lundsberg, Anders Björkman, Nebojsa Malesevic, Christian Antfolk

**Affiliations:** 1 Department of Biomedical Engineering, Faculty of Engineering, Lund University, Lund, Sweden; 2 Department of Hand Surgery, Institute of Clinical Sciences, Sahlgrenska Academy, University of Gothenburg and Sahlgrenska University Hospital, Gothenburg, Sweden

**Keywords:** Electromyography, Motor unit depth, Motor unit spatial distribution, Motor unit localization, Biomedical engineering, Motor control

## Abstract

The spatial distribution of muscle fibre activity is of interest in guiding therapy and assessing recovery of motor function following injuries of the peripheral or central nervous system. This paper presents a new method for stable estimation of motor unit territory centres from high-density surface electromyography (HDsEMG). This completely automatic process applies principal component compression and a rotatable Gaussian surface fit to motor unit action potential (MUAP) distributions to map the spatial distribution of motor unit activity. Each estimated position corresponds to the signal centre of the motor unit territory. Two subjects were used to test the method on forearm muscles, using two different approaches. With the first dataset, motor units were identified by decomposition of intramuscular EMG and the centre position of each motor unit territory was estimated from synchronized HDsEMG data. These positions were compared to the positions of the intramuscular fine wire electrodes with depth measured using ultrasound. With the second dataset, decomposition and motor unit localization was done directly on HDsEMG data, during specific muscle contractions. From the first dataset, the mean estimated depth of the motor unit centres were 8.7, 11.6, and 9.1 mm, with standard deviations 0.5, 0.1, and 1.3 mm, and the respective depths of the fine wire electrodes were 8.4, 15.8, and 9.1 mm. The second dataset generated distinct spatial distributions of motor unit activity which were used to identify the regions of different muscles of the forearm, in a 3-dimensional and projected 2-dimensional view. In conclusion, a method is presented which estimates motor unit centre positions from HDsEMG. The study demonstrates the shifting spatial distribution of muscle fibre activity between different efforts, which could be used to assess individual muscles on a motor unit level.

## Introduction

Mapping the distribution of motor unit activity is essential for understanding basic muscle neurophysiology. Motor units are the smallest functional parts of voluntary movement, consisting of a single motor neuron and the muscle fibres it innervates. These muscle fibre groups directly relay information from the nervous system. Peripheral or central nervous system injuries may therefore affect the distribution of motor unit activity in muscles. Tracking how motor unit activity is distributed could provide a quantifiable assessment of the severity of an injury and to what extent lost motor function is recovered, tools which are in high demand^1^. Furthermore, it could guide treatment of neuromuscular disorders such as spasticity^2^. The in vivo study of motor units is predominantly done with intramuscular and surface electromyography (EMG)^3–6^. More recent studies have explored motor unit imaging using ultrafast ultrasound^7^, as well as a combination of ultrasound and EMG^8–11^.

Intramuscular EMG (iEMG) provides high spatial selectivity and therefore reliable identification of motor units, which can be done either manually or through various algorithms^12–14^. Due to this high spatial selectivity of iEMG, the identified motor units are located close to the recording electrode. By knowing the position of the recording electrode, the position of the motor units is known as well. However, the high spatial selectivity and the invasive nature of the recording limit the ability of iEMG to simultaneously study many motor units. Thus, making it difficult to acquire a broader view of the distribution of motor unit activity since this would require a large number of intramuscular electrodes, which carries the risk of damage to muscle fibres, infection, as well as discomfort for the patient.

Ultrafast ultrasound is a promising alternative to iEMG, where decomposition of velocity images is used to single out individual motor units; a technique which directly identifies motor unit positions. However, decomposition of ultrafast ultrasound requires further validation, especially for many active motor units when higher muscle force is used. An additional shortcoming of ultrafast ultrasound is the intense computational and data storage requirements for such measurement systems, which limits the duration of recordings.

Surface EMG (sEMG), on the other hand, is a well-studied non-invasive technique. In contrast to iEMG, electrodes on the skin surface need to be less spatially selective since they are further from the active muscle. Consequently, the signal is less discernible and the individual motor units cannot be identified manually. To compensate for this, high-density sEMG (HDsEMG) is used with more advanced signal separation techniques, in order to decompose the data into motor unit activity^15–18^. These decomposition techniques identify spike trains, which are the time instances of motor unit action potentials (MUAPs), from individual motor units. The MUAP is a compound signal of the synchronized single fibre action potentials of a motor unit firing. Thus, the contribution of each single fibre is not discernible with these techniques. Furthermore, the shape of individual MUAPs in a motor unit are not directly identified. Instead, each motor unit is represented by an average MUAP, generated using the identified spike train. The average contribution of all MUAPs from one motor unit to the recorded signal at each surface electrode is in this paper referred to as the surface MUAP distribution. Averaging the MUAP signals across each motor unit firing is referred to as spike-triggered averaging. The motor unit activity, however, is identified without positional information. Inferring motor unit positions from surface EMG therefore requires additional techniques.

Approaches to mapping muscle activity from sEMG have been proposed for many years using finite elements^19–21^, which has been further used to study the motor unit distribution in stroke survivors^22^. A faster and simpler approach has also been proposed and iterated upon using an analytical volume conductor model, and a curve fit to the peak amplitudes of the surface MUAP distribution^23–28^. This curve fit approach estimates a motor unit’s depth from the full width at half maximum (FWHM) of the surface potential peak amplitudes, perpendicular to an assumed muscle fibre direction. The FWHM, estimated with the spread of a Gaussian fit, has been shown to correlate with motor unit depth^23^. The motor unit depth and the point along the skin surface with the largest peak amplitude, in both the temporal and spatial domain, provide coordinates for each motor unit. Since MUAPs are compound signals from multiple single fibres, the estimated motor unit coordinates describe a centre point, with the largest peak amplitude, on an equivalent fibre that would generate the same potential distribution. The equivalent fibre’s position is assumed to be a weighted sum of individual single fibre positions. However, distinctions are not made between different sets of weights or distributions of individual fibres, which is a limitation of the method. Furthermore, this curve fitting approach is done with a fit in one dimension^23–28^, by placing the electrodes over the largest peak amplitude of the surface MUAP distribution perpendicular to the muscle fibre direction, which are both unknown prior to analysis of the signal. Thus, the coordinate along the fibre direction is unclear, and different fibre rotations from anatomical differences and muscle fibre pinnation may affect the depth estimate. Additionally, using a single linear array of electrodes makes the approach highly susceptible to noise at individual channels. HDsEMG may, however, solve these limitations with the additional information from a large number of channels.

The motor unit localization approach presented in this paper expands upon the original curve fit concept by using a two-dimensional elliptical Gaussian fit. This approach enables automatic localization of motor units with different surface amplitude peaks and fibre directions, as well as being robust to noise. In a pilot study, the presented method is first tested on a dataset of simultaneous intramuscular and surface EMG of the forearm, containing three intramuscular fine wire electrodes and an 8-by-8 surface electrode grid. Motor units are identified by decomposition of iEMG, with positions estimated from the synchronized HDsEMG data and compared to the positions of the fine wire electrodes, with depths measured using ultrasound. The presented method is then tested on a dataset consisting of only HDsEMG, using two adjacent 13-by-5 surface electrode grids. Motor units are identified by decomposition of HDsEMG, and the spatial distributions of motor units are qualitatively assessed for different efforts.

## Methods

### Data acquisition

Two separate tests of the method are presented with two participants. Simultaneous intramuscular and surface EMG was recorded with additional ultrasound images taken before the EMG recordings on the first participant, and only surface EMG was recorded on the second participant. Both participants were right-handed and neurologically intact. The participants provided informed consent, and the study was approved by the Regional Ethical Review Board in Lund, Sweden (DNR 2017-297). The motor unit localization method uses the shape of surface MUAP distributions across the skin to estimate motor unit positions. The identification of surface MUAP distributions is done differently for the two tests and thus separated in this section. The localization method is the same in both tests and thus presented in one section after the “Data acquisition” section.

#### Motor units from synchronized iEMG

**Figure 1:**
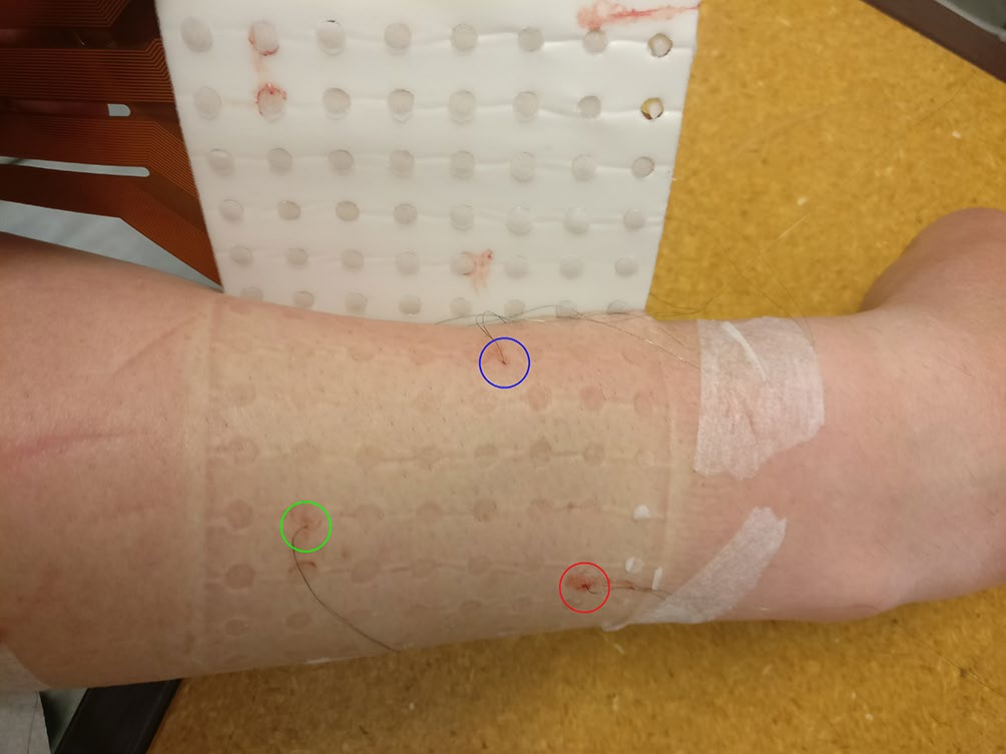
A post-measurement image showing the three fine wire electrodes inserted into the extensor indicis proprius (red circle), extensor pollicis longus (green circle), and abductor pollicis longus (blue circle) of the participant’s posterior forearm. For illustrative purpose, the HDsEMG grid is elevated. During the recordings, this grid is placed on top of the forearm, covering the fine wire electrodes.

For the first dataset, three intramuscular paired fine wire electrodes (0.051 mm in diameter stainless steel wires with 2 mm uninsulated ends, Chalgren, Gilroy, USA) were inserted into the right forearm of the participant with an insertion needle, targeting the following muscles: the extensor indicis proprius (EIP), extensor pollicis longus (EPL), and abductor pollicis longus (APL) (Fig. 1). At the time of insertion, the depths of the insertion needles were measured using an ultrasound machine (EPIQ 7, Phillips, The Netherlands with a linear transducer L18-5 at 9 MHz centre frequency). The insertion needles were measured because the fine wires electrodes were not visible on the ultrasound image. After the ultrasound measurements were performed, a HDsEMG electrode array, of 64 electrodes in an 8 × 8 differential configuration with 10 mm interelectrode distance (Model ELSCH064NM3, OT Bioelettronica, Torino, Italy), was placed on the skin on top of the intramuscular fine wire electrodes (Fig. 1). Differential signals were obtained by recording the difference between each electrode and the following closest electrode in the grid in the proximal to distal direction. All EMG data were recorded at a sampling rate of 10,240 Hz, amplified 150 times, and analog bandpass filtered between 0.7 and 4400 Hz, with a 16-bit analog-to-digital converter (Quattrocento, OT Bioelettronica, Torino, Italy). The sampling rate and analog filtering was the same for iEMG and HDsEMG data as a result of recording simultaneously with the same system. However, this did not affect the data since further digital filtering would be applied to the HDsEMG data regardless. The efforts used for this recording were index finger flexion and extension, thumb flexion and extension, and thumb palmar adduction and abduction, corresponding to the muscles targeted with iEMG. The arm was placed in an isometric force rig^29^ with 90-degree lateral rotation from the prone position, with visual force feedback displayed in a LabVIEW (National Instruments, Austin, Texas, USA) program as a guide for consistent finger muscle contractions. After determining the maximum voluntary contraction (MVC) force for each effort, the participant performed multiple isometric agonist–antagonist contractions by following a sinusoidal force curve (including both directions) with an amplitude of 20% MVC. See^29^ for a detailed description. All forces were recorded concurrently with the EMG recordings, which enabled the selection of EMG data only when force was applied in the selected direction to minimize crosstalk from other muscles.

For this first test, motor unit spike trains were only identified from the iEMG data (fine wire electrodes), which had known positional references from the ultrasound measurements. Two-pass zero-phase filtering was performed on the iEMG data using a third order Butterworth notch filter with cutoff frequencies at 49 and 51 Hz, and bandpass filter with cutoff frequencies at 1 Hz and 2000 Hz. For the HDsEMG data, two-pass zero-phase filtering was performed using a third order Butterworth notch filter with cutoff frequencies at 49 and 51 Hz, and bandpass filter with cutoff frequencies at 5 Hz and 500 Hz. Motor unit spike trains were then identified from the filtered iEMG data by peak detection and high-dimensional density-based spatial clustering of peaks described in Ref.^18^ with the DBSCAN algorithm^14^. The DBSCAN algorithm groups data points into clusters using a distance parameter and a minimum neighbours parameter. Data points with sufficient neighbours within the distance parameter are labelled as core points, and data points with too few neighbours but within range of a core point are labelled as edge points. Distinct groups of core points and edge points form separate clusters, and remaining data points not in range of any core points are labelled as outliers. As input to DBSCAN, a window of 100 samples (approximately 10 ms) was used as unique variables, generating a single high-dimensional data point, for each peak. The distance parameter, was set between 0.5 and 0.6 and the minimum neighbours parameter was set to 50. These parameters were chosen empirically, after manually optimizing the clustering for the smallest number of false positives. Spike trains were not identified for the HDsEMG data in this test. Instead, each spike train from the iEMG data was synchronized with the HDsEMG data, and spike-triggered averaging was used to calculate the surface MUAP distributions, later used for motor unit localization. The window size for spike-triggered averaging was 400 samples (approximately 40 ms).

#### Motor units from decomposition of HDsEMG

**Figure 2:**
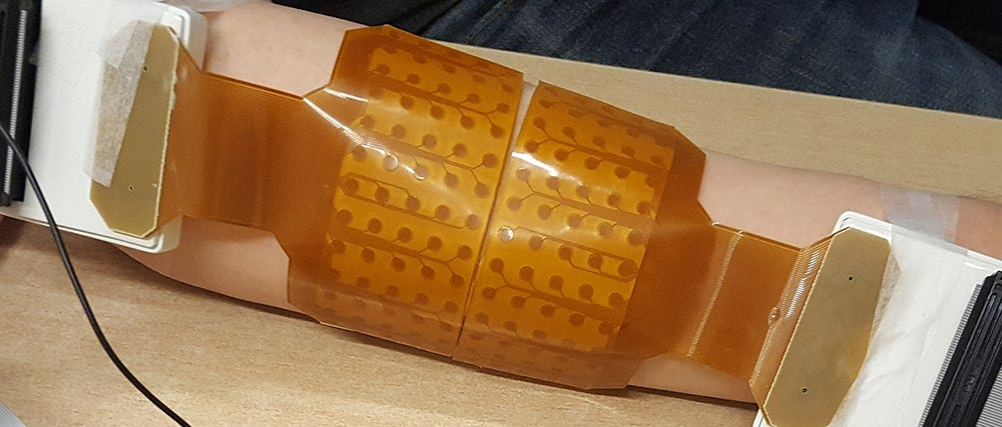
An image of two adjacent 13 × 5 electrode grids placed on the posterior side of the forearm.

For the second dataset, two HDsEMG electrode arrays, both 64 electrodes in a 5 × 13 monopolar configuration with 8 mm interelectrode distance (Model ELSCH064NM2, OT Bioelettronica, Torino, Italy), were placed adjacently on the right forearm of the second participant, resulting in a 10 × 13 grid (Fig. 2). The efforts used for the second test were wrist extension, wrist extension with radial deviation, index finger extension, ring finger extension, little finger extension, thumb palmar abduction, and thumb palmar adduction. EMG data was recorded for 60 s for each effort at a sampling rate of 2048 Hz, amplified 150 times, and analog bandpass filtered between 0.7 and 900 Hz, with a 16-bit analog-to-digital converter (Quattrocento, OT Bioelettronica, Torino, Italy). The forearm was in a prone position for all efforts except wrist extension with radial deviation, where the wrist was laterally rotated approximately 45° from the prone position, to isolate the extensor carpi radialis muscles. Isometric contractions were performed by applying a constant force to a vertically placed force gauge (Mark-10 model M5-20). Constant set force levels were used, at 1 N for finger and thumb contractions and 2 N for wrist contractions. At set force levels, the number of active motor units is assumed to be relatively consistent, which facilitates the comparison of the spatial distributions of motor unit activity between efforts. Using force levels at a percentage of MVC for each effort is commonly used to adjust for differences in muscle strengths. However, this test aims to distinguish the spatial distribution of motor unit activity between different wrist and finger contractions; and changing the force levels would introduce an additional factor to consider which affects the motor unit distribution. Thus, the force and number of motor units is kept as consistent as possible.

For this second test, motor units were identified by decomposition of the HDsEMG data, without known positional references. Two-pass zero-phase filtering was performed using a third order Butterworth notch filter with cutoff frequencies at 49 and 51 Hz, and bandpass filter with cutoff frequencies at 5 Hz and 500 Hz. The filtered HDsEMG data was decomposed with the iterative peel-off method described in Ref.^18^. This decomposition method estimates individual motor unit spike trains using the Fast Independent Component Analysis algorithm^30^ while accounting for time structures by extending the dataset with delayed copies similar to other methods^15–17^. The spike-triggered average motor unit signal is removed from the HDsEMG dataset in each iteration^18^, allowing iterative discovery of more motor units. Decomposition was run for 30 iterations with an extension factor of 6, each delayed by 2 samples, for each effort. Spike-triggered averaging then generated the surface MUAP distributions used for motor unit localization. Motor units were removed manually if their surface distributions were too medially or laterally positioned relative to the HDsEMG grid, since the surface fit applied in this method would not properly cover those areas. This positional requirement is a limitation of the current method to be considered.

### Localization

**Figure 3:**
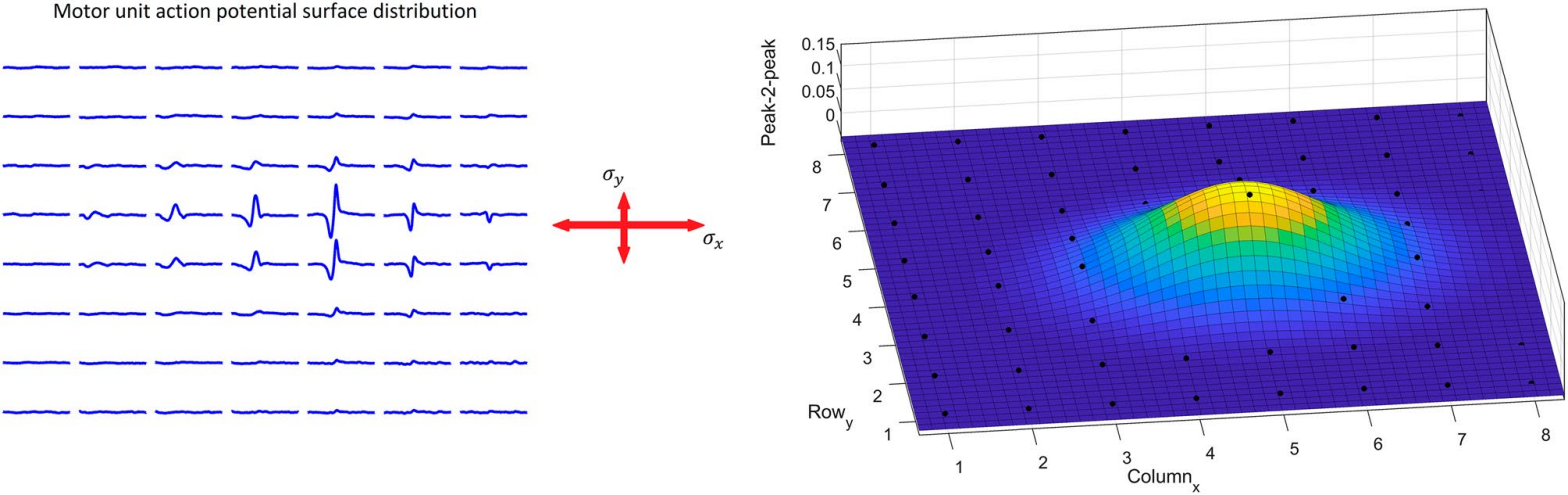
The localization method applies a general Gaussian surface fit to the peak-to-peak amplitudes of the surface MUAP distribution. The largest spread, ${\upsigma }_{{\text{x}}}$, indicates the fibre direction, whilst ${\upsigma }_{{\text{y}}}$ indicates the spread perpendicular to the fibre direction, which relates to motor unit depth.

The localization method presented in this section was applied principally in the same manner in both tests, with differing model parameters accounting for differences in forearm shape, electrode layout, and recording configuration. The positions of motor units were estimated using a Gaussian surface fit to the peak-to-peak amplitudes of the surface MUAP distribution (Fig. 3), for both differential signals (first test) and monopolar signals (second test). The surface MUAP amplitude is assumed to decay the fastest perpendicular to the muscle fibre direction from the electrode closest to the motor unit centre, and subsequently decays the slowest along the muscle fibre direction. As such, the surface MUAP distribution provides information on both the position and the fibre direction of each motor unit.

#### Principal component compression as noise reduction

As a pre-processing step, surface MUAP distributions were first smoothened by principal component compression. The estimated surface MUAP distributions occasionally contain residual noise, due to, e.g., errors in the original spike train, or from a low number of firings where spike-triggered averaging has not cancelled out all the noise. The idea of this step is that by choosing the amount of compression correctly, then mainly components for random noise are removed, while components for the surface MUAP amplitudes of interest remain^18^. In this paper, surface MUAP distributions were compressed down to a matrix of four samples per channel, by matrix multiplication with four eigenvectors of the covariance matrix corresponding to the four largest eigenvalues, and then resized with the transposed eigenvector matrix. The compression was important to ensure that noise at channels far from the motor unit would not dominate the lower amplitudes and subsequently impair the fitting process. Without compression, noise at these distant channels was found to often generate large errors for the surface fit, which would then converge poorly.

#### Gaussian surface fit

For a HDsEMG grid, each electrode’s column and row define the variables $x$ and $y$. However, the muscle fibres are not always aligned with the $x$ and $y$ axes of the electrode grid. In order to apply a Gaussian surface fit to the surface MUAP distribution, the potential offset can be accounted for by introducing a rotation angle. The generalized rotatable Gaussian function is derived by introducing a rotation matrix to the standard two-dimensional Gaussian function.

1 $$\begin{array}{c}f\left(\widehat{x},\widehat{y}\right)=A\cdot \exp\left(-\left(\frac{{\left(\widehat{x}-{\widehat{x}}_{0}\right)}^{2}}{2{\sigma }_{x}^{2}}+\frac{{\left(\widehat{y}-{\widehat{y}}_{0}\right)}^{2}}{2{\sigma }_{y}^{2}}\right)\right),\end{array}$$

$$\begin{array}{c}\left[\genfrac{}{}{0pt}{}{\widehat{x}}{\widehat{y}}\right]=\left[\begin{array}{cc}{\text{cos}}\theta & -{\text{sin}}\theta \\ {\text{sin}}\theta & {\text{cos}}\theta \end{array}\right]\left[\begin{array}{c}x\\ y\end{array}\right]=\left[\begin{array}{c}x{\text{cos}}\theta -y{\text{sin}}\theta \\ x{\text{sin}}\theta +y{\text{cos}}\theta \end{array}\right].\end{array}$$

After simplification, resulting in

2 $$\begin{array}{c}f\left(x,y\right)=A\cdot {\text{e}}{\text{x}}{\text{p}}\left(-\left(a{\left(x-{x}_{0}\right)}^{2}+2b\left(x-{x}_{0}\right)\left(y-{y}_{0}\right)+c{\left(y-{y}_{0}\right)}^{2}\right)\right),\end{array}$$

$$a=\frac{{{\text{cos}}}^{2}\theta }{2{\sigma }_{x}^{2}}+\frac{{{\text{sin}}}^{2}\theta }{2{\sigma }_{y}^{2}},$$

$$b=-\frac{{\text{sin}}2\theta }{4{\sigma }_{x}^{2}}+\frac{{\text{sin}}2\theta }{4{\sigma }_{y}^{2}},$$

$$c=\frac{{{\text{sin}}}^{2}\theta }{2{\sigma }_{x}^{2}}+\frac{{{\text{cos}}}^{2}\theta }{2{\sigma }_{y}^{2}},$$

where $A$ is the peak amplitude of the Gaussian function at the centre $({x}_{0}, {y}_{0})$, with the spread $({\sigma }_{x}, {\sigma }_{y})$ and rotation angle $\theta$. These six coefficients are estimated by fitting the function to the peak-to-peak values of each electrode in the surface MUAP distribution, using least squares, with the curve fitting toolbox in MATLAB (MathWorks, Natick, Massachusetts, USA). Then, it is assumed that the largest spread of the distribution is along the fibre direction; and thus, the smallest $\sigma$ defines the spread perpendicular to the fibre direction (Fig. 3). The spread perpendicular to the fibre direction and the rotation angle are then used to estimate the motor unit depth in a cylindrical volume conductor model.

#### Volume conductor model

The conductor model in this method is an analytical homogenous cylindrical single-layer model, which uses the Gaussian fit’s amplitude centre, spread, and rotation to calculate the final motor unit position. Since the calculations are performed in the plane perpendicular to the fibre direction, the model is assumed to be isotropic. Although advanced multilayer conductor models have been developed^31,32^, a single-layer model was used in this paper since additional layers were found to overcomplicate the method and generate too many unknown parameters. These choices are further explored in the discussion section.

Previous approaches have used a power function to model signal decay with increased distance^23^, from the laws of electrostatics which assumes that the dynamics of the system are negligible. However, the MUAP signal is generated by the temporary displacement of ions and subsequent return flow to a neutral state, which is intuitively similar to the generation of waves. In this paper, signal strength, $V$, is instead modelled akin to attenuation of waves using Bouguer–Lambert–Beer’s extinction law, where the amplitude decreases exponentially with distance, $d$, in an absorbing medium with a factor for attenuation strength, $Q$.

3 $$\begin{array}{c}V={V}_{0}{e}^{-Qd}.\end{array}$$

This model choice is justified both in terms of physical plausibility and ease of calculation. The exponential decay model assumes an internal starting amplitude, ${V}_{0}$, at the motor unit territory centre, where $d=0$. A power function, such as ${d}^{-Q}$, grows infinitely for $d\to 0$, which makes it difficult to estimate an internal starting amplitude. The exponential function provides similar decay properties to the power function, although it tends faster towards 0. Ultimately, both models are simplifications with different assumptions and could therefore both be viable. The attenuation strength is unknown, and the optimal value may differ between individuals, muscles, and recording configurations. A higher attenuation value ultimately produces deeper estimates of motor unit positions. However, a shift in attenuation strength offsets the estimates of all motor units and has only a minor impact on their relative position to other motor units. For the first test, $Q$ was estimated post hoc, by calibrating for one of the three fine wire electrodes. The optimal value of $Q$ was identified for motor units found in the iEMG signal in the APL muscle, producing a mean of ${\mu }_{Q}=1.36$ with a standard deviation ${\sigma }_{Q}=0.16$ from two motor units. Since the first dataset was recorded in a differential configuration, the same attenuation factor was not applicable to the second dataset recorded in a monopolar configuration. Due to the lack of ground truth, the attenuation factor for the second test was set to $1$ for simplicity.

**Figure 4:**
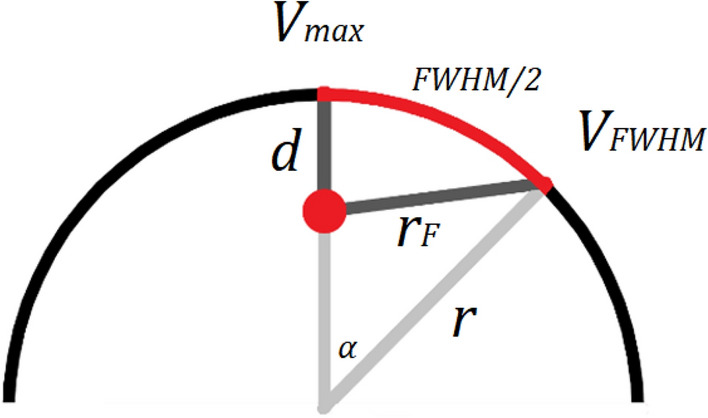
An illustration of the geometry and values used in the model to estimate motor unit depth.

The ratio between the peak of the Gaussian distribution and the point at FWHM is, by definition, 2. From this, the depth of the motor unit, $d$, and radial distance to the surface position at half maximum surface potential, ${r}_{F}$, (Fig. 4) are related by

4 $$\begin{array}{c}\frac{{V}_{max}}{{V}_{FWHM}}=\frac{{V}_{0}{e}^{-Qd}}{{V}_{0}{e}^{-Q{r}_{F}}}\Rightarrow {\text{ln}}2=Q\left({r}_{F}-d\right),\end{array}$$

5 $$\begin{array}{c}{r}_{F}=d+{\text{ln}}2/Q.\end{array}$$

Roeleveld et al.^23^ previously described the geometric relation between motor unit depth and radial distance. As a result of the cosine rule, a second expression for radial distance is generated, where the radial distance creates a triangle with the radius $r$, and $r-d$, with the opposing angle $\alpha =FWHM/2r$.

6 $$\begin{array}{c}{{r}_{F}}^{2}={r}^{2}+{\left(r-d\right)}^{2}-2r\left(r-d\right){\text{cos}}\alpha .\end{array}$$

The FWHM relates to the spread of the Gaussian fit perpendicular to the fibre direction according to

7 $$\begin{array}{c}FWHM=2\sqrt{2{\text{ln}}2}\cdot \sigma .\end{array}$$

After combining the two expressions for radial distance, the depth is directly calculated from

8 $$\begin{array}{c}d=\frac{2{r}^{2}{\text{cos}}\alpha -2{r}^{2}+{\left({\text{ln}}2/Q\right)}^{2}}{2\left(r{\text{cos}}\alpha -r-{\text{ln}}2/Q\right)}.\end{array}$$

This, however, only applies to motor units where the fibre direction is aligned with the cylindrical model. For other fibre directions, the effective radius of the expression changes.

#### Effective radius

The radius of the cylinder was obtained by measuring the circumference of the forearm for both participants. The radius values were $48 \, {\text{mm}}$ for the first dataset, and $50 \, {\text{mm}}$ for the second dataset. For motor units with a rotation angle, $\theta$, the radius value in Eqs. (6) and (8) increases, since the calculation is done perpendicular to the fibre direction in a cylindrical model. For example, in the extreme case with a fibre direction in the transverse plane of the cylinder, the depth is calculated with the surface amplitude distribution along the flat surface in the proximal to distal direction on the cylinder. Thus, the effective radius is the radius value used for calculation of motor unit depth, which increases with increased rotation angle. The measured radius, $r$, is related to the effective radius, ${r}_{eff}$, by making use of projections for two circle sectors, sharing the corner ${V}_{max}$. For a rotated fibre direction, imagine the circle sector spanning ${V}_{max}$, ${V}_{FWHM}$, and the centre point, with the two radii ${r}_{eff}$ and the arc $FWHM/2$. The radius from the centre point to ${V}_{FWHM}$ projects onto the other radius with the cosine of the angle given by the arc $FWHM/2$. This will be a common point, or depth, between the circle sectors, due to the cylindrical shape of the model. The second circle sector is perpendicular to the cylinder direction and makes use of the measured radius, $r$, and the corner ${V}_{max}$. For this second circle sector, the projection of the radii lands on the common point, or depth, when the arc is $FWHM/2\cdot {\text{cos}}\theta$. The distance between the common point and ${V}_{max}$ in these two cases generate the equation

9 $$\begin{array}{c}r-r{\text{cos}}\alpha ={r}_{eff}-{r}_{eff}{\text{cos}}\beta ,\end{array}$$

$$\alpha =\frac{FWHM\cdot {\text{cos}}\theta }{2r}, \beta =\frac{FWHM}{2{r}_{eff}}.$$

By approximation of the cosine function, we get a simplified equation for the effective radius.

10 $$\begin{array}{c}{\text{cos}}x\approx 1-{x}^{2}/2,\end{array}$$

11 $$\begin{array}{c}\Rightarrow r-r\left(1-\frac{{FWHM}^{2}{{\text{cos}}}^{2}\theta }{8{r}^{2}}\right)={r}_{eff}-{r}_{eff}\left(1-\frac{{FWHM}^{2}}{8{r}_{eff}^{2}}\right),\end{array}$$

12 $$\begin{array}{c}{r}_{eff}=r/{{\text{cos}}}^{2}\theta .\end{array}$$

With the surface fit parameters, the estimated attenuation strength, and the effective radius value, the centre position of each motor unit is estimated. While the spread of the surface fit perpendicular to the fibre direction informs motor unit depth, the spread along the fibre direction could conceivably inform motor unit fibre length. However, this possibility is not explored in this paper. Instead, fibre lengths are plotted as half the spread along the fibre direction, for visualization purposes only.

### Ethics approval and consent to participate

The study was approved by the Regional Ethical Review Board in Lund, Sweden (DNR 2017-297) and was conducted in accordance with the tenets of the Declaration of Helsinki. All participants were informed about the contents of the experiments, both verbally and in writing, and gave their informed and written consent.

## Results

### Motor units from synchronized iEMG

**Figure 5:**
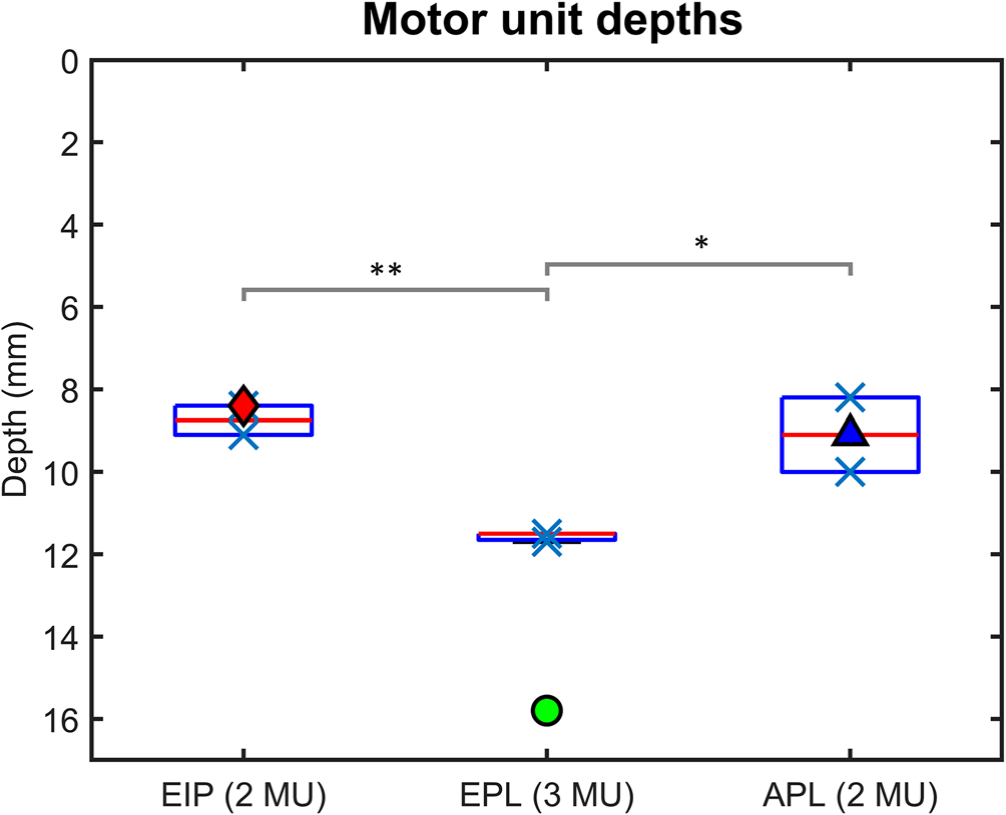
A boxplot of the estimated depths, grouped by muscle, from 7 surface MUAP distributions from one participant. The blue crosses indicate the estimated depth of each motor unit centre. The motor units were first identified with iEMG, then temporally matched with the HDsEMG data. The red, green, and blue shapes represent the depth of each fine wire insertion needle, measured by ultrasound. One and two asterisks indicate a significant difference at $\mathrm{\alpha }=0.05$ and $\mathrm{\alpha }=0.01$ respectively. The blue triangle overlaps with the red median line for motor units from the APL since the two motor units from this fine wire electrode were used to estimate the attenuation factor of the model.

**Figure 6:**
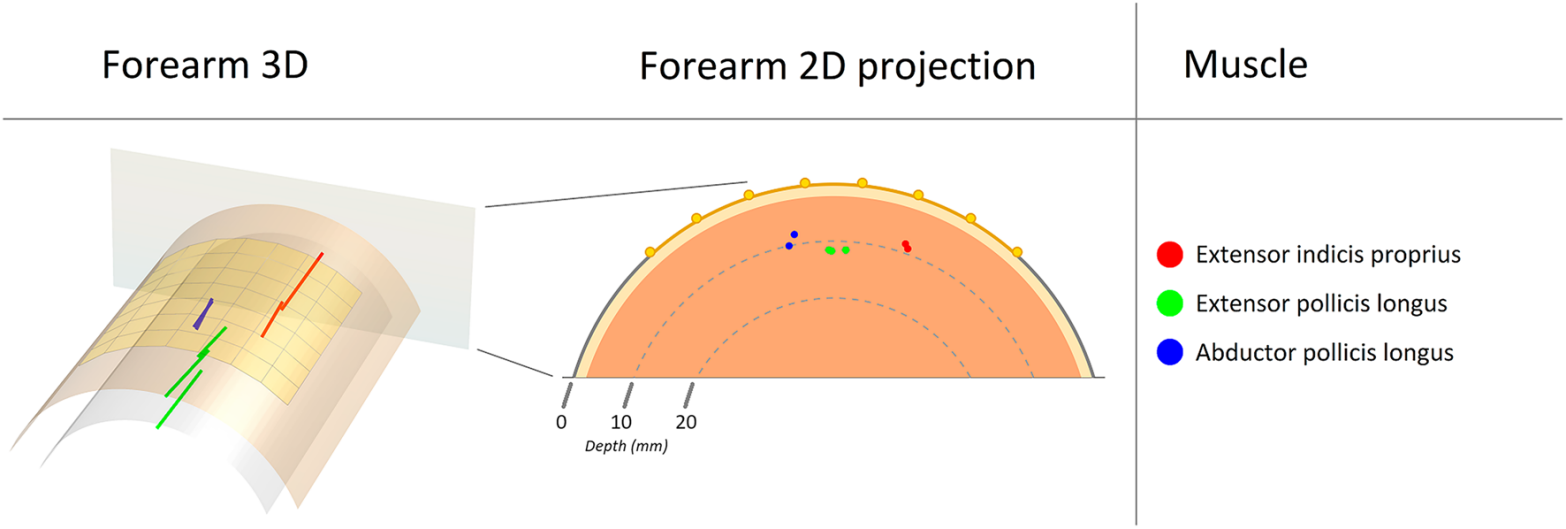
The left figure shows the visualization of 7 motor units in 3D in a cylindrical model. The top surface represents the skin and the electrode grid, and the underlying concentric layer indicate 10 mm depth. A projection onto the transverse plane is shown in the right figure where 10- and 20-mm depths are marked. The yellow layer illustrates subcutaneous fat; however, this distinction is not included in the model. The motor unit centres, indicated by coloured dots, were identified from iEMG and their positions estimated from synchronized HDsEMG data, for the extensor indicis proprius (red), extensor pollicis longus (green), and abductor pollicis longus (blue), in the right forearm of the participant.

From the three intramuscular fine wire electrodes in the first participant, a total of 7 motor units were identified and temporally matched with the HDsEMG data, generating 7 surface MUAP distributions. From these surface MUAP distributions, the estimated depth of each motor unit is shown in Table 1 and grouped by muscle in a boxplot in Fig. 5. The depth of the needles inserting the intramuscular fine wire electrodes were 8.4, 15.8, and 9.1 mm for the extensor indicis proprius (EIP), extensor pollicis longus (EPL), and abductor pollicis longus (APL) respectively. The mean estimated depth for motor units from each wire was 8.7, 11.6, and 9.1 mm, with standard deviations 0.5, 0.1, and 1.3 mm, for the EIP, EPL, and APL, respectively. The resolution of these estimates is further addressed in the discussion section. Visualization of the motor unit positions in 3D is shown in Fig. 6, alongside a projection of the motor unit centres onto the transverse plane. The motor units are clearly localized to the insertion points on the skin surface (seen in Fig. 1). However, the difference in estimated motor unit depth between the muscles of the participant was not as large as the difference between the insertion depths of the fine wire electrodes measured by ultrasound. Thus, it is possible that the model underestimates differences in depth. It may also be the result of the displacement of muscles under tension, or a displacement of the fine wire electrodes after insertion. While these estimated differences were less than in the ultrasound reference, motor units from the EPL muscle still produced the deepest estimates (Fig. 5). For one-tailed two-sample t-tests, the mean motor unit depth of the EPL muscle was 2.8 mm greater than the EIP muscle with $p = 0.001$, and 2.5 mm greater than the APL muscle with $p = 0.018$. After Bonferroni correction for multiple comparisons is applied to these values, the results are significant at the $\alpha =0.01$ and $\alpha =0.05$ levels respectively. However, strong conclusions from statistical analysis should be avoided, due to the low number of motor units in the test which limits this study.

**Table 1:** The estimated depth of motor units from surface MUAP distributions, identified by temporally matching iEMG and HDsEMG data from one participant. The two motor units from the APL were used as calibration points for the attenuation factor of the model.

Muscle	Motor unit depth (mm)	Insertion needle depth (mm)
EIP	9.1	8.4
	8.4	
EPL	11.5	15.8
	11.5	
	11.7	
APL	8.2	9.1
	10

### Motor units from decomposition of HDsEMG

**Figure 7:**
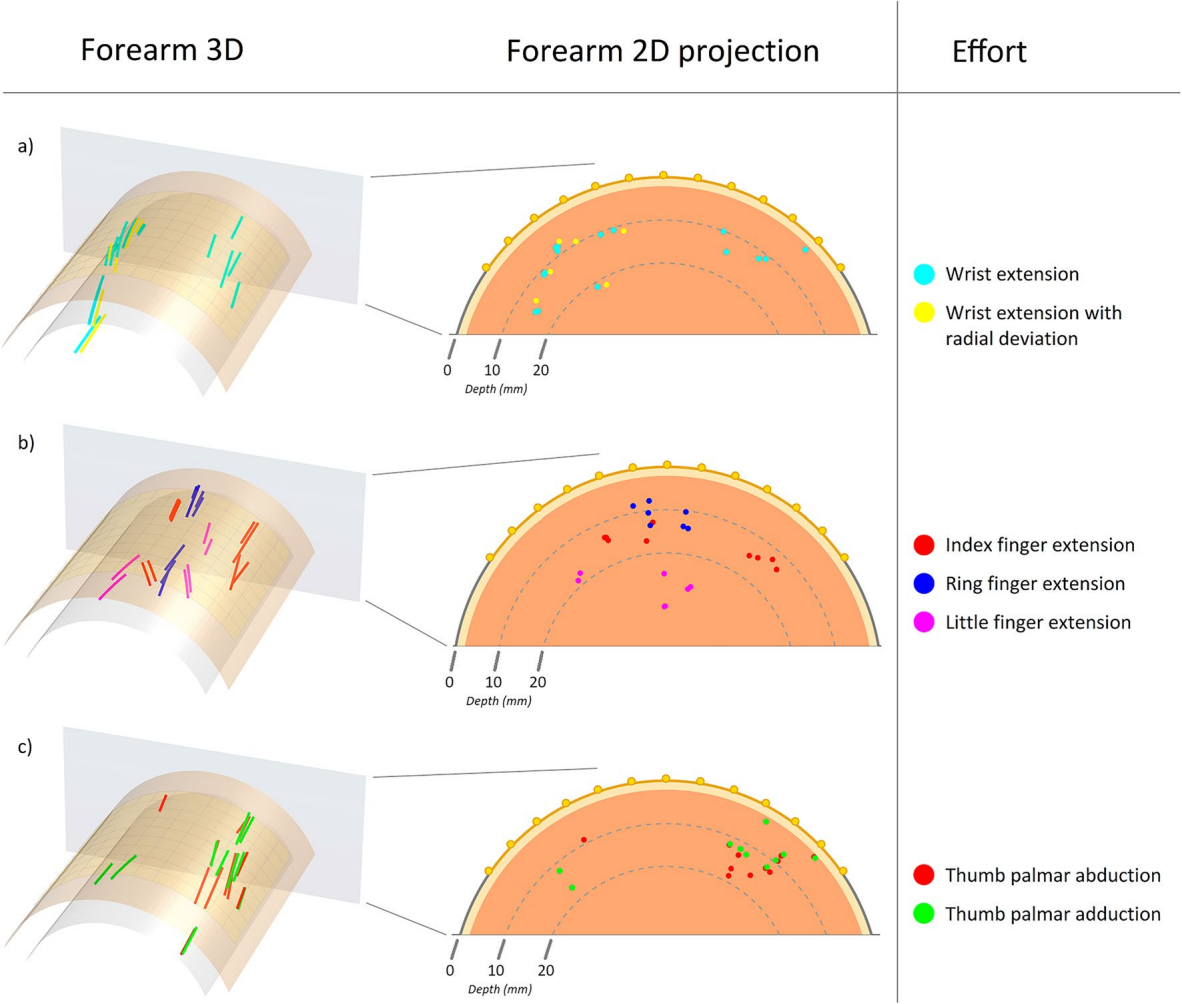
The figures in the left column show the visualization of motor units in 3D in a cylindrical model. The top surface represents the skin and the electrode grid, and the underlying layer indicate 10 mm depth. A projection onto the transverse plane is shown in the right column where 10- and 20-mm depths are marked. The yellow layer illustrates subcutaneous fat; however, this distinction is not included in the model. The motor unit centres, indicated by coloured dots, were identified by decomposition of HDsEMG for 7 separate efforts. The results are grouped into wrist extensions (**a**), finger extensions (**b**), and thumb efforts (**c**) to aid analysis.

From HDsEMG decomposition of the seven different efforts, a total of 79 motor units were identified. After discarding 14 motor units that were too far outside the bounds of the HDsEMG grids, the remaining 65 motor units were used for the localization method (Table 2). Visualization of the motor unit positions in 3D is shown in Fig. 7, alongside a projection of the motor unit centres onto the transverse plane. To aid analysis, the seven efforts were grouped into wrist efforts, finger efforts, and thumb efforts. For wrist extension there were two clear areas of motor unit activity, at the radial and ulnar edges of the grid, which provides a preliminary suggestion that these are the areas of the extensor carpi radialis brevis or longus (ECRB and ECRL) and the extensor carpi ulnaris (ECU) respectively (Fig. 7a). When wrist extension was performed with radial deviation, the motor unit activity on the ulnar side disappeared, which suggests that an isolated contraction with the ECR muscles was successfully made. Additionally, the direction of these radial fibres aligns with the expected anatomy of the ECR muscles, as seen in the 3D plot. For finger extensions, the motor unit activity was mainly identified in between the previous estimates of the ECR muscles and the ECU muscle, suggesting this is the area of the extensor digitorum communis muscle (Fig. 7b). However, additional activity on the ulnar side can be seen for index finger extension, which overlaps with the previous estimate of the ECU muscle, which may indicate some amount of stabilizing synergistic contraction. The estimates for little finger extension are less convincing due to their pronounced depth. This depth could be the result of either displacement of the muscles under tension, synergistic contractions of the deeper abductor and extensor pollicis longus muscles (APL and EPL), or an error in the depth estimates specific to these motor units. For thumb efforts, the activity distributions were the least conclusive (Fig. 7c). The large amount of overlapping activity on the ulnar side for both thumb palmar abduction and adduction suggests that these are not motor units of the APL or EPL muscles but could instead be stabilizing contractions of the wrist. Further tests across the forearm are needed to identify the area of, e.g., abductor pollicis longus.

**Table 2:** The number of identified motor units and the vertical upward force applied to the force gauge for each of the 7 efforts.

Effort	Motor units	Force (N)
Wrist extension	15	2
Wrist extension with radial deviation	6	2
Index finger extension	9	1
Ring finger extension	7	1
Little finger extension	7	1
Thumb palmar abduction	11	− 1
Thumb palmar adduction	10	1

## Discussion

In this study, a new automatic method was developed for non-invasive estimation of motor unit positions, consisting of principal component compression and a custom surface fit applied to peak-to-peak amplitudes of surface MUAP distributions, as well as a volume conductor model with exponential signal attenuation. Surface MUAP distributions have been thoroughly studied^23,24,28,33,34^, demonstrating the relationship between motor unit depth and the FWHM of the amplitudes of the surface MUAP distribution. Thus, this paper focuses on providing a method which improves upon previous methods^23–27^. The new method presented in this paper is automatic and carries a very low computational load, in contrast to, e.g., finite element methods^19–21^. This enables the estimation of a large number of motor units, required for properly mapping the spatial distribution of motor unit activity. However, it is limited to motor units within the confines of the HDsEMG grid. For end applications, it is furthermore important to consider the limitations of decomposition algorithms, and the number of motor units which can reliably be identified. The new method is flexible as it is applicable to different motor unit positions and fibre directions. This was exemplified by the wrist extensions in the second test (Fig. 7a), which demonstrated the methods ability to identify the slightly diagonal direction of the ECR muscles. In contrast, previous studies used linear arrays of electrodes or selected a single column of electrodes from a grid manually^22–27,33,35^, which requires prior knowledge of motor unit positions and fibre directions for correct estimations.

A method was recently proposed with generalized electrode placements which identifies surface amplitude maps and the centre of gravity of surface MUAP distributions, using normalized peak-to-peak values, generated via spike-triggered averaging on a monopolar HDsEMG grid^36^. However, their method only identifies motor unit positions at the surface and does not include the depth of motor units. Still, this centre of gravity approach highlights the potential asymmetries in surface MUAP distributions when contrasted with the peak amplitude centre. The Gaussian fit is rigid in this regard, and accounting for such asymmetries is a potential next step in improving the method presented in this paper. Furthermore, asymmetries in the surface MUAP distribution could perhaps be used inform fibre rotation in the depth direction. Better models and estimates for fibre length should also be explored by making use of, e.g., the spread along the fibre direction.

The stability of motor unit estimates is improved by using a grid of electrodes, rather than a linear array. Due to the high number of electrodes, the method is less affected by individual poor channels, improving stability. Furthermore, principal component compression reduces possible residual noise after spike-triggered averaging. This ensures that the Gaussian fit is applied only to the surface MUAP signal, reducing the risk of converging on poor local minima determined by residual noise. In addition, the presented method includes a new correction for the effective radius of a cylindrical model for fibre directions at an angle. However, the extent to which this correction affects the results was not determined and should be studied further.

The placement of the HDsEMG grid is very important, as was seen in both tests. In the first test, the surface MUAP distributions of the EIP were close to the distal edge of the grid, which may impact the accuracy of the Gaussian fit. In the second test, it is possible that the APL and EPL muscles were too distal in relation to the grids to be easily detected, which could explain the resulting motor unit distributions (Fig. 7c). Future studies should ensure full coverage of the muscles by targeting muscles closer together or using a larger array.

Furthermore, the recording configuration affects the surface MUAP distribution and thus the estimated FWHM^23,33^. For differential recordings, the surface MUAP distribution sometimes contains a row of low amplitudes where the MUAP signal shifts from increasing to decreasing amplitude. This occurs when the peak amplitude of the motor unit signal lies in the middle between two electrodes. The electrodes are then affected equally by the MUAP, resulting in a low differential signal between them. This may negatively affect the Gaussian fit and increases the risk of converging on an erroneous local minimum. A monopolar configuration might therefore be more suitable for this method. Monopolar surface MUAP distributions generally provide larger FWHM values^23,33^, which results in deeper motor unit estimates. Thus, a smaller attenuation factor may be used to compensate for this effect. Whether depth discrimination between motor units is affected should be studied further. Additionally, depth estimates could be affected by the size of the motor unit territories. A larger spread of neuromuscular junctions may result in a larger FWHM, which could be conflated with increased depth.

The estimation of the attenuation factor in the conductor model is one of the main limitations of the current method. In the first test, an attenuation factor was identified by calibrating for one of three fine wire electrodes. This value may not be generalizable to other muscle groups or individuals. Since the forearm contains many closely packed muscles, it presents a good test for localization and muscle discrimination. However, future studies should include a range of muscle groups and many subjects. Furthermore, the depth values in this paper are reported with 0.1 mm resolution. This choice was made due to the small differences in depth estimates for the motor units from the EPL muscle, resulting in a standard deviation of 0.1 mm. However, it is important to note that more data is required to properly determine the precision of the method. Additionally, while the positions of the insertion needles were precisely obtained from ultrasound images, it is not known to what extent the fine wire electrodes move after insertion. Using ultrasound with better resolution to identify the position of the fine wire electrodes after removing the insertion needle would greatly improve the reliability of iEMG as reference points. Conceivably, iEMG could be used once per patient in future applications to determine the attenuation factor, with subsequent recordings done with only HDsEMG. The use of iEMG, however, is preferably avoided due to patient discomfort.

The choice of conductor model, with an exponential attenuation instead of a power function, was briefly motivated in “Volume conductor model” section but should be explored further. The Bouger–Lambert–Beer’s extinction law used in this study is an empirical law concerning the attenuation of light through a material. We model the MUAP as a voltage source, generating an electromagnetic wave, propagating through the tissue. However, since the fluctuations of the potential field from the flow of ions is relatively slow, the application of the extinction law may be limited. A power function derived from electrostatic laws could still be a better model for MUAP amplitudes, which is a potential limitation of this study. However, neither an exponential nor a power function is a complete description of the MUAP source, as cylindrical muscle fibres. Due to the high degree of simplification, validity is difficult to assess from a theoretical point. As such, we argue that performances should be assessed and compared for multiple models in an end application. While a power function may work well to describe potential amplitudes along the skin surface, it is difficult to generate estimates for an internal starting amplitude, since the distance in these functions cannot be set to zero, unlike in the exponential function. Such internal estimates were not done in this study. A future study could therefore explore this, by comparing the internal estimate from surface EMG, ${V}_{0}$ in Eq. (3), with intramuscular EMG at a known depth. The thickness of the subcutaneous layer varies across muscle groups and should be studied to determine whether it affects the estimates enough to limit the utility of the method. Since the conductor model in this paper is only a single-layer model, the properties of the different layers are approximated to a single attenuation factor. More complex multilayered models^31,32^ could hypothetically provide better depth discrimination between motor units across muscle groups, by making use of more parameters. However, it is also possible that shifting the attenuation factor in a single-layer model can sufficiently account for varying thicknesses of the subcutaneous layers, which should be explored. The advantage of the single-layer model is in the simplicity of implementation and would be the preferred choice if viable. Furthermore, the calculations assume that the muscle is isotropic in the cross-sectional plane. The isotropic assumption is only made in the plane perpendicular to the fibre direction, since the length of the surface MUAP distribution in the fibre direction is not used for depth estimation. It is possible that the conductivity along the fibre direction could influence the distribution width as well, however, this effect was not assessed and assumed to be negligible.

## Conclusion

A new automatic method for position estimation of motor units has been presented, based on a rotatable Gaussian surface fit applied to surface MUAP distributions. The method is tested using simultaneous iEMG and HDsEMG as well as only HDsEMG. The method provided distinct areas of activity associated with different muscles and demonstrated the shifting spatial distribution of motor unit activity between different efforts. This information could be used to aid non-invasive assessments of individual muscles on a motor unit level.

## Data Availability

The datasets used and/or analysed during the current study are available from the corresponding author on reasonable request.
